# Compensatory Intercellular Mitochondrial Transfer Improves Bioenergetics in P301L Tau-Affected Neuronal Cells

**DOI:** 10.3390/cells15121101

**Published:** 2026-06-17

**Authors:** Aurélien Riou, Aline Broeglin, Andreas Papassotiropoulos, Anne Eckert, Amandine Grimm

**Affiliations:** 1 Cell Biology & Energy Metabolism, University Psychiatric Clinics (UPK) Basel, University of Basel, 4002 Basel, Switzerland; aurelien.riou@unibas.ch (A.R.); aline.broeglin@unibas.ch (A.B.); 2 Research Cluster Molecular and Cognitive Neurosciences, Department of Biomedicine, University of Basel, 4055 Basel, Switzerland; andreas.papas@unibas.ch; 3 Neurobiology Laboratory for Brain Aging and Mental Health, University Psychiatric Clinics (UPK) Basel, University of Basel, 4002 Basel, Switzerland; anne.eckert@unibas.ch

**Keywords:** intercellular mitochondrial transfer, mitochondria, tauopathies, astrocytes, neurons

## Abstract

**Highlights:**

**What are the main findings?**

- Intercellular mitochondrial transfer from astrocytes to neurons is significantly increased in the presence of P301L mutant tau.
- Contact-dependent transfer appears to be the dominant pathway of mitochondrial transfer, with an increased number of tunneling nanotubes between astrocytes and P301L-expressing neuronal cells.
- Abnormal tau protein does not significantly alter the intracellular fate of transferred mitochondria.
- Transferred astrocytic mitochondria remain functional and improve cellular bioenergetics in recipient cells.

**What are the implications of the main findings?**

- Our findings identified a novel protective mechanism supporting neuronal bioenergetics in tauopathies.
- Mitochondrial transfer may represent an attractive therapeutic strategy to compensate for energy deficits in neurodegeneration.

**Abstract:**

Tauopathies are a group of neurodegenerative diseases characterized by the accumulation of abnormal tau protein, leading to mitochondrial dysfunction. Because of neurons’ high energy demands, such impairments significantly contribute to neuronal vulnerability. Recent evidence indicates that mitochondria can be transferred between cells to support energy-deficient cells through intercellular mitochondrial transfer (IMT). Given the impact of pathological tau on mitochondrial transport and cytoskeletal dynamics, we hypothesized that IMT is altered in tauopathies. We investigated IMT from astrocytes to neurons, as well as the influence of abnormal tau protein on this process, using co-cultures of SH-SY5Y cells (neuronal model) and A172 cells (astrocytic model). Key data were then confirmed in human iPSC-derived neurons and astrocytes. We show that IMT is enhanced in the presence of abnormal tau and occurs predominantly through contact-dependent mechanisms. Transferred mitochondria were either integrated into the host mitochondrial network, degraded in lysosomes, or remained isolated in the recipient cells’ cytosol. This transfer improved cellular respiration and was associated with increased bioenergetics in pathological cells. Together, our results highlight IMT as a link between tau pathology and neuronal metabolic adaptation, suggesting that this process reflects an endogenous metabolic adaptation holding therapeutic potential to mitigate energy deficits in neurodegenerative diseases.

## 1. Introduction

The brain consumes approximately 20% of the body’s energy, although comprising only 2% of total body mass, to sustain its functions [1]. To meet this high energy demand, the brain relies on mitochondria, essential organelles in all eukaryotic cells, due to their critical role in adenosine triphosphate (ATP) production. This production occurs via oxidative phosphorylation, the primary energy-generating pathway in cells [2]. While mitochondria are classically defined as the powerhouses of the cells, recent advances highlight their role as dynamic signaling hubs that regulate critical non-energetic processes in the brain, including inter-organelle communication, vesicular trafficking, and immunomodulation[3]. Consequently, disruptions in these homeostatic and signaling pathways are now recognized as key drivers of neurological disease pathology alongside bioenergetic deficits. Indeed, multiple studies have demonstrated that mitochondria are impaired in neurodegenerative diseases, including tauopathies, thereby contributing to disease progression [4,5].

Tauopathies encompass a heterogeneous group of neurodegenerative disorders (such as Alzheimer’s disease, Pick’s disease, corticobasal degeneration, progressive supranuclear palsy, or frontotemporal lobar degeneration), characterized by the accumulation of abnormal tau protein [6]. Clinically, these diseases manifest with a broad spectrum of neurological deficits, including cognitive and psychiatric disorders, motor dysfunctions, and difficulties with speech, swallowing, and vision [7]. Unfortunately, no cure has been identified for these conditions yet. Physiologically, tau is a member of the microtubule-associated protein family [1]. It is predominantly expressed in neurons, especially within axons, where it plays a pivotal role in stabilizing microtubules by binding to tubulin and promoting its polymerization. Consequently, tau is essential for the axonal transport of organelles, neuronal plasticity, axon growth, elongation, morphogenesis, differentiation, and neurite polarity [1,8,9]. In tauopathies, tau becomes abnormally hyperphosphorylated, leading to its aggregation, causing microtubule destabilization and, ultimately, neuronal dysfunction and death [10]. In addition, a prominent hallmark of all these tau-related diseases is the impairment of brain bioenergetics and mitochondrial function [11,12]. Indeed, abnormal tau protein is known to impair almost all mitochondrial functions, including mitochondrial transport [13,14], dynamics [15], bioenergetics [4,5], and mitophagy [16], thereby contributing to neuronal degeneration. However, the precise molecular mechanisms by which tau affects mitochondrial function remain elusive.

Recently, studies on intercellular mitochondrial transfer (IMT) have emerged, demonstrating this mechanism across various organs and pathological contexts [17,18]. Studies have shown that healthy cells can transfer mitochondria to support dysfunctional cells, while damaged cells may pass defective mitochondria to healthy cells for degradation and recycling [2]. IMT has been observed in various cell types, including astrocytes, mesenchymal stem cells, and immune cells, highlighting its broad physiological and pathological significance. Two primary mechanisms for intercellular mitochondrial transfer have been identified:

- Extracellular vesicles (EVs): EVs are bilayer membranous structures secreted into the extracellular space. Astrocytes have been shown to shed EVs containing functional mitochondria [19].
- Tunneling Nanotubes (TNTs): TNTs are membrane channels composed of F-actin that connect two or more cells. This channel allows the transfer of cytoplasmic molecules or organelles from one cell to another [20].

Furthermore, other mechanisms may be possible, such as extracellular mitochondria internalization by recipient cells or transfer via gap junctions [21].

Astrocytes are well known for their crucial supportive role in the central nervous system, particularly for neurons. They are known to regulate the neuronal microenvironment by providing metabolic substrates, clearing oxidative stress, and modulating synaptic activity [22,23,24]. Given their essential support functions and substantial mitochondrial pool, despite their primarily glycolytic metabolism [25], astrocytes have been studied for their involvement in IMT and identified as key players in mitochondrial transfer to neurons [19,26,27,28].

Evidence showed that IMT between astrocytes and neurons has beneficial effects on the brain, such as increasing ATP levels and neuronal viability in recipient cells in brain injury [19,26,27]. Mitochondrial transport and function are known to be negatively impacted in the context of tauopathies [1,29]. However, no study has explored whether abnormal tau protein impacts IMT between neurons and astrocytes. Determining whether astrocytes can transfer healthy mitochondria to neurons affected by tau pathology could provide critical insights into neuroprotection and potential therapeutic strategies. Therefore, this study investigated whether mitochondrial transfer occurs between astrocytes and neurons in the presence of abnormal tau protein, and the impact of this transfer on the recipient cells. To address this question, we used healthy SH-SY5Y cells (control cells) and SH-SY5Y cells overexpressing the P301L tau mutation as a neuronal model, in co-culture with an astrocytic cell line (A172 cells). The impact of abnormal tau on IMT was also confirmed using co-cultures of iPSC-derived neurons bearing the P301L mutation and iPSC-derived astrocytes. We aimed to identify which transfer pathway was involved and to determine the fate of the transferred mitochondria in recipient cells.

## 2. Materials and Methods

### 2.1. Cell Lines

In our study, we used the human neuroblastoma cell line SH-SY5Y (#CRL-2266, ATCC, Manassas, VA, USA) as our neuronal model. As a specific model of tauopathy, SH-SY5Y cells stably overexpressing the P301L mutation in the MAPT gene (chromosome 17) and tagged with green fluorescent protein (GFP) were employed (P301L cells). This cell line was developed using lentiviral gene transfer and kindly provided by Professor Jürgen Götz (Queensland Brain Institute, University of Queensland, Australia) [29,30]. As a control, we used SH-SY5Y cells expressing the GFP-vector alone (control).

To investigate IMT, the human glioblastoma cell line A172 (#CRL-1620, ATCC, Manassas, VA, USA) was used as our astrocytic model for the co-culture with the neuronal cell lines.

Routine monitoring for mycoplasma contamination was performed on all cell cultures using the MycoAlert^®^ PLUS Mycoplasma Detection Kit (#LT07-710, Lonza Bioscience, Basel, Switzerland). Experiments involving cell lines were conducted using cells between passages 10 to 20.

### 2.2. Cell Culture and Differentiation

Both neuronal and astrocytic cell lines were cultured in Dulbecco’s Modified Eagle Medium (DMEM) (#D6429, Sigma-Aldrich, St. Louis, MO, USA) supplemented with 1% (*v*/*v*) penicillin/streptomycin (#4-01F00-H, Bioconcept, Allschwil, Switzerland), 1% GlutaMax (#35050061, ThermoFisher Scientific, Waltham, MA, USA), 10% of heat-inactivated fetal bovine serum (FBS) (#35-015-CV, Corning, NY, USA). In addition, the SH-SY5Y cell culture medium was supplemented with 5% heat-inactivated horse serum (#2-05F00-I, Bioconcept, Allschwil, Switzerland). To maintain stable expression of the P301L mutation and fluorescent tags, the culture medium was supplemented every two passages with 4.5 μg/mL of blasticidin (#Ant-bl-1, InvivoGen, Toulouse, France) for the GFP-tagged cells, while 500 μg/mL of Geneticin (#SC-29065B, Santa Cruz Biotechnology, Dallas, TX, USA) was used for cells stably expressing mitochondria-labeled RFP, or BFP2 tags. The cells were cultured in 10 cm^2^ dishes, split twice a week, and replated upon reaching approximately 80% confluence.

Prior to the experiments, SH-SY5Y-GFP cells were differentiated according to the following protocol. The day after plating, the culture medium was replaced with DMEM supplemented with 1% (*v*/*v*) penicillin/streptomycin, 1% GlutaMax, 1% non-essential amino acid (#11140-035, Gibco, Waltham, MA, USA), 3% FBS, and 10 µM of retinoic acid (#04454002, ThermoFisher Scientific, Waltham, MA, USA). After forty-eight hours, the medium was replaced with fresh DMEM supplemented with 1% (*v*/*v*) penicillin/streptomycin, 1% GlutaMax, 1% FBS, and 10 µM of retinoic acid. Twenty-four hours later, the medium was changed to Neurobasal medium (#21103049, ThermoFisher Scientific, Waltham, MA, USA) supplemented with 1% (*v*/*v*) penicillin/streptomycin, 1% of Glutamax, 1% of B27 (#17504044, ThermoFisher Scientific, Waltham, MA, USA) and 50 ng/mL of BDNF (#450-02-100UG, PeproTech, Hamburg, Germany). The cells were then incubated in this medium for 48 h before co-culture. Forty-eight hours prior to the co-culture, the A172 cells were placed in Neurobasal medium supplemented with 1% (*v*/*v*) penicillin/streptomycin, 1% Glutamax, and 2% B27.

### 2.3. Culture and Differentiation of iPSC-Derived Neurons and Astrocytes

Findings were further validated using human MAPT P301L/WT mutant iPSCs (#F0510.2, called “P301L” condition), and the isogenic CRISPR-Cas9-edited wild-type MAPT WT/WT iPSCs (#F0510.2d2E7, called “Control” condition), kindly provided by Dr. Celeste Karch and the Tau Consortium Stem Cell Group [30]. This model was used to confirm the experimental outcomes observed in the co-cultures of the immortalized cell line.

Cells were maintained in Essential 8™ (E8) Medium containing Essential 8™ Supplement (#A1517001, Gibco, Waltham, MA, USA) and 1% penicillin/streptomycin with daily media exchanges and passaged twice per week using TrypLE Select (#12604013, ThermoFisher Scientific, Waltham, MA, USA).

Neural induction of iPSCs to neural progenitor cells (NPCs) was performed in three sequential phases on Geltrex-coated plates (A1413202, ThermoFisher Scientific, Waltham, MA, USA). All phases used a basal induction medium composed of Advanced DMEM/F12 (#12634028, ThermoFisher Scientific, Waltham, MA, USA), 1% P/S, 2 mM Glutamax, and 1% B27. During phase 1 (days 1 to 7), the basal medium was supplemented with 10 μM SB-431542 (#S-7800, LucernaChem, Luzern, Switzerland), 1 μM LDN-193189 (#HY-12071, MedChem Express, Monmouth Junction, NJ, USA), and 2 μM XAV939 (#2848932, LucernaChem, Luzern, Switzerland). During phase 2 (days 8 to 14), medium was switched to basal medium containing 200 nM LDN-193189 and 2 μM XAV939. For the last phase, the NPC population was expanded and cultured in basal medium supplemented with 20 μg/mL FGF-2 (#CYT-557, LucernaChem, Luzern, Switzerland). Throughout induction, media were refreshed daily, and cells were passaged using TrypLE Select at confluency. NPC quality was rigorously monitored via immunocytochemistry to ensure consistent expression of SOX1, SOX2, Nestin, and PAX6.

Then, NPCs were differentiated into mature neurons on Geltrex-coated plates using a two-stage media protocol. In the first seven days, cells were cultured in Neuronal Differentiation Medium I (ND I) (Neurobasal with 1% P/S, 2 mM Glutamax, 1% B27, and 5 μM DAPT (#HY-13027, MedChem Express, Monmouth Junction, NJ, USA). Once proliferation stopped, NPCs were plated on PLO/laminin-coated dishes (40,000 cells/cm^2^) with ND II (ND I components supplemented with 200 μM L-ascorbic acid (#A7506, Sigma-Aldrich, St. Louis, MO, USA) 1.8 mM CaCl_2_ (#449709, Sigma-Aldrich, St. Louis, MO, USA) and 20 ng/mL BDNF(#450–02, PeproTech, Hamburg, Germany) until day 28, with bi-weekly media exchanges. By day 28, successful maturation was confirmed through the expression of B3TUB and MAP2 markers (Figure S1).

In parallel, NPCs were differentiated into mature astrocytes on Geltrex-coated surfaces (initial density 50,000 cells/cm^2^) using a two-stage protocol. For the first 14 days, NPCs were maintained in astrocyte induction medium, consisting of DMEM/F12 supplemented with 1% P/S, 1% Glutamax, 1% B27, 1% N_2_, 10 ng/mL EGF (Sigma-Aldrich, E9644) and 10 ng/mL LIF (PeproTech, 300–05). Subsequently, the cultures were transitioned to maturation medium (DMEM/F12 with 1% P/S, 1% Glutamax, 1% B27, and 20 ng/mL CNTF (#450–13, PeproTech, Hamburg, Germany) until Day 42. Media were refreshed daily from Day 0 to 28 and every three days thereafter. At confluency, cells were passaged using TrypLE Select and re-seeded at a density of 30,000–40,000 cells/cm^2^. On Day 42, astrocyte maturity was validated by immunocytochemistry using GFAP, GLAST, and S100B (Figure S1).

### 2.4. Lentivirus Preparation and Cell Transduction

To study mitochondrial transfer from astrocytic to neuronal cells, we used transduced cells expressing GFP tags and fluorescent mitochondria. For this purpose, we employed custom-designed lentiviral vectors from VectorBuilder (Chicago, IL, USA). These vectors can be identified using the following IDs, which provide detailed information on the VectorBuilder website (http://www.vectorbuilder.com).

1. Green Fluorescent Protein (GFP)-labeled cells: pLV[Exp]-Bsd-CMV>EmGFP (ID: VB231221-1477jqh).
2. Mock vector: pLV[Exp]-CMV>ORF_Stuffer (ID: VB900140-2612kkt).
3. Red Fluorescent Protein-labeled mitochondria (mitoRFP): pLV[Exp]-CMV>{COX8 presequence:TagRFP-T}-{SV40_promoter}>Neo (ID: VB230714-1048hhg).
4. Blue Fluorescent Protein 2-labeled mitochondria (mitoBFP2): pLV[Exp]-CMV>{COX8 presequence:TagBFP2}-{SV40_promoter}>Neo (ID: VB240506-1616nnn).

SH-SY5Y and A172 cell lines were transduced at a multiplicity of infection of 5, while iPSC-derived neurons and astrocytes were transduced at a multiplicity of infection of 10, following the manufacturer’s instructions. Transduction efficiency was subsequently validated via fluorescence imaging.

### 2.5. Direct and Indirect Co-Culture

Direct (contact-dependent) and indirect co-cultures (non-contact-dependent) using a 3 µm mesh insert (#9313002, cellQART, Northeim, Germany) were established at a 1:1 neuronal-to-astrocytic cell ratio. Two time points were tested with a co-culture incubation time of 24 or 48 h for the SH-SY5Y and A172 cell lines, and iPSC-derived neurons and astrocytes were directly co-cultured for 48 h only for IMT validation.

### 2.6. Tunneling Nanotubes Counting

To quantify TNT numbers, GFP-expressing SH-SY5Y cells (control or P301L) were co-cultured with A172 cells for 48 h and subsequently fixed with 4% paraformaldehyde (#P6148-50G, CAS: 30525-89-4, Sigma-Aldrich, St. Louis, MO, USA) for 10 min. To distinguish TNTs from neurites in co-culture, actin filaments and βIII-tubulin were stained as follows. Cells were permeabilized with 0.15% Triton X-100 (#T8787, Sigma-Aldrich, St. Louis, MO, USA) (in PBS) for 15 min, followed by blocking with 2% bovine serum albumin (BSA; in PBS) for 1 h to prevent non-specific antibody binding. Cells were then incubated overnight at 4 °C with mouse anti-βIII-tubulin (#MAB1195, R&D Systems, Zug, Switzerland) diluted 1:1000 and protected from light. After three washes, cells were incubated with goat anti-mouse IgG Alexa Fluor 568 secondary antibody (#ab175473, Abcam, Cambridge, UK) (1:1000) for 1 h. In parallel, actin filaments were stained with phalloidin-iFluor 647 (#ab176759, Abcam, Cambridge, UK) (1:1000) for 1 h according to the manufacturer’s instructions. Cells were washed three times, and slides were mounted using antifade mounting medium (#H-1000, Vector Labs, Newark, CA, USA).

Images were acquired using a Nikon Eclipse Ti2 fluorescence microscope (Nikon, Tokyo, Japan) and deconvolved with Huygens software (version 24.10.0; Scientific Volume Imaging B.V., Hilversum, The Netherlands). Fields of view were selected based on the proximity between neuronal and astrocytic cells to ensure the presence of potential cell–cell interactions. TNTs were blindly and manually quantified using FIJI software (version 2.16.0). Only actin-based structures connecting a neuronal cell to an astrocytic cell and lacking colocalization with βIII-tubulin staining were counted as a TNT.

### 2.7. Microscopy and Colocalization Analysis

After the co-culture on precoated collagen coverslips (#GC-18-1.5, Neuvitro, Vancouver, WA, USA), cells designated for fluorescence microscopy were fixed with 4% paraformaldehyde for 10 min at room temperature and washed three times with PBS. Slides were then mounted using an antifade mounting medium. Images were captured using a Nikon Eclipse Ti2 fluorescence microscope and deconvolved using Huygens software.

To determine the fate of the transferred mitochondria, we performed a 48 h direct co-culture between GFP-tagged SH-SY5Y (control or P301L) expressing also mitoRFP, and A172 expressing mitoBFP2. After co-culture, cells assigned to the mitophagy-induced condition were treated overnight with antimycin A (4 µM) and oligomycin (10 µM) (from the Seahorse XFp CellMito Stress Test Kit, #103010-100, Agilent Technologies, Basel, Switzerland) to depolarize mitochondria and thereby enhance mitophagic activity. Lysosomes were stained with LysoTracker Deep Red (100 nM, 1 h) (#L12492, Invitrogen, Carlsbad, CA, USA) prior to fixation with 4% paraformaldehyde for 10 min.

Colocalization analyses were then conducted to determine whether astrocytic mitoBFP2 mitochondria were integrated into the mitoRFP neuronal mitochondrial network or targeted to lysosomes (LysoTracker). Quantification was performed in FIJI (version 2.16.0) using the JACoP plugin and based on Manders’ coefficients. To avoid false-positive signals, colocalization values below 5% were considered negligible and classified as no colocalization.

To generate 3D representations of each channel, false colors were used with the surface creation tool in Imaris (version 9.9.1, Oxford Instruments, Oxford, UK). For each channel, surfaces were manually defined based on the visible signal in the z-stack images, following the structures observed by eye to outline the regions of interest.

### 2.8. Flow Cytometry & Cell Sorting

Flow cytometry experiments were performed to quantify IMT from astrocytic cells expressing mitoRFP to GFP-tagged neuronal cells (control and P301L). We used an unstained mock control co-culture and fluorescence-minus-one (FMO) controls to determine the optimal voltages, compensation parameters, and gating strategy (Figure S2). After 24 or 48 h of direct and indirect co-culture, cells designated for flow cytometry analysis were dissociated into single cells using Accutase (#ICTAT104, Chemie Brunschwig, Basel, Switzerland) and resuspended in HBSS (#14065049, Gibco, Waltham, MA, USA). To eliminate potential cell aggregates, the suspension was passed through a 40 µm mesh filter (#43-10040-60, pluriSelect, Leipzig, Germany). Before analysis on the BD LSRFortessa flow cytometer, dead cells were stained with DAPI (Sigma-Aldrich, #MBD0015) at 1 µg/mL concentration. Data analysis was performed using Flowjo software (version 10.10.0, BD Biosciences, Franklin Lakes, NJ, USA) to assess the presence of astrocytic mitoRFP in GFP-positive neuronal cells.

Using the same protocol, cell sorting was performed with the CytoFLEX SRT Sorter. (Beckman Coulter Life Sciences, Indianapolis, IN, USA) SH-SY5Y cells were separated into two populations: those containing astrocytic mitochondria and those without. The sorted cells were then plated in Neurobasal medium supplemented with 1% (*v*/*v*) penicillin/streptomycin, 1% GlutaMAX, and 15% FBS to later assess the effects of transferred mitochondria on recipient cell function, namely on ATP production and cellular respiration.

### 2.9. ATP Assay

After the sorting of SH-SY5Y cells containing astrocytic mitochondria and those without astrocytic mitochondria, cells were plated into a 96-well plate (20,000 cells/well). After 24 h, ATP levels were assessed using the “ATPlite 1-Step Luminescence Assay System” kit (#6016943, Revvity Health Sciences Inc., Waltham, MA, USA), following the manufacturer’s instructions. The substrate was added directly to the wells, and bioluminescence was measured using the Cytation 5 plate reader (Agilent Technologies, Basel, Switzerland) within 20 min of reagent addition. The luminescence signals from each condition were compared with an ATP standard curve (0 to 5 µM) to determine the corresponding ATP concentrations. All the data were normalized based on the living cell area assessed using the CellTracker Blue CMAC dye (5 µM) (#C2110, Invitrogen, Carlsbad, CA, USA).

### 2.10. Mitochondrial Membrane Potential Assay

Mitochondrial membrane potential (MMP) was assessed 24 h after seeding isolated neuronal cells (with or without transferred astrocytic mitochondria) into a 96-well plate (20,000 cells per well). MitoTracker Deep Red, a dye that accumulates in mitochondria in a membrane-potential-dependent manner, was used to measure MMP change between the SH-SY5Y cells that received or did not receive astrocytic mitochondria [31]. After 45 min of incubation with MitoTracker Deepred (1 µM) (#M22426, Invitrogen, Carlsbad, CA, USA), cells were washed, and fluorescence intensity was read at ex: 644 nm/em: 665 nm using the Cytation 5 plate reader. Data were normalized with CellTracker Blue CMAC (5 µM).

### 2.11. Respiration Assay

Cellular respiration was continuously monitored using the Resipher device (Lucid Scientific, Atlanta, GA, USA), which enables real-time measurement of the oxygen consumption rate (OCR) directly within the cell incubator [32]. The device was magnetically attached to a sensor-equipped lid placed on 96-well plates containing 20,000 isolated cells per well. The experimental recording was initiated immediately after the cells were sorted (SH-SY5Y cells with or without astrocytic mitochondria) and plated. OCR values were extracted using the Lucid Scientific software (https://lab.lucidsci.com/#/experiments; accessed on 14 April 2026) platform and normalized to the living cell area determined with CellTracker™ Blue CMAC (5 µM).

### 2.12. Statistical Analysis

Statistical analyses were performed using GraphPad Prism software (version 10.4.1, GraphPad Software Inc., San Diego, CA, USA). All quantified data were presented as mean ± standard deviation (SD). Data were subjected to a Grubbs’ test to identify and remove significant outliers (Figure S2 highlights the outliers that were removed in specific experiments). Normality was assessed using the Shapiro–Wilk test. For comparisons between two groups, a Welch’s *t*-test was applied when data followed a normal distribution, whereas a Mann–Whitney test was used when normality was not met. For comparisons involving more than two groups or multiple factors, two or three-way ANOVA was performed as appropriate. A *p*-value of less than 0.05 was considered statistically significant, with significance levels denoted as follows: *p* < 0.05 (*), *p* < 0.01 (**), and *p* < 0.001 (***).

## 3. Results

### 3.1. Mitochondrial Transfer Occurs from Astrocytes to Neurons in Both Healthy and P301L-Expressing Cells

**Figure 1:**
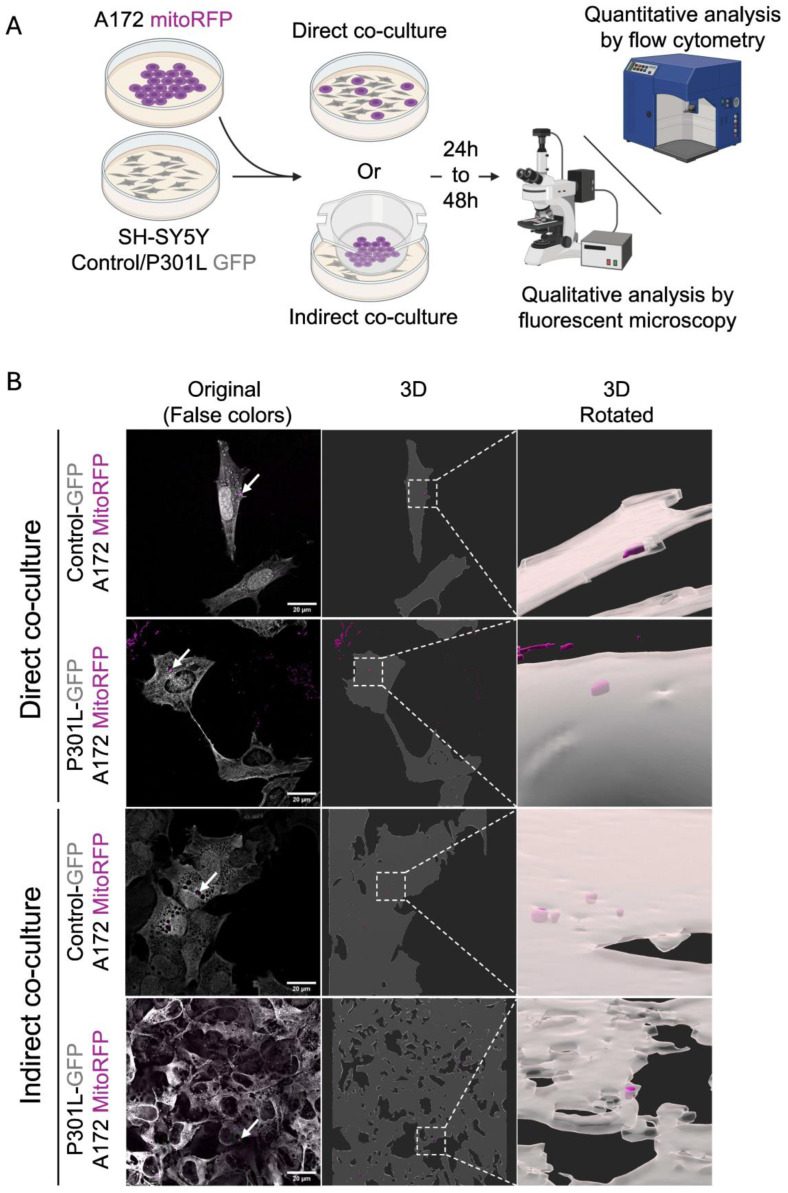
Qualitative assessment of mitochondrial transfer. (**A**) Experimental overview: To investigate mitochondrial transfer from astrocytic A172 cells expressing RFP-tagged mitochondria (A172 mitoRFP) to GFP neuronal SH-SY5Y cells (SH-SY5Y GFP), co-culture experiments were performed either directly (1:1 ratio in the same dish) or indirectly using a 3 µm mesh insert. Mitochondrial transfer was assessed qualitatively by fluorescence microscopy and quantitatively by flow cytometry. The figure was made with Biorender Riou, A. (2026) https://BioRender.com/5ii2vtf (accessed on 11 June 2026). (**B**) After 24 h of co-culture, mitoRFP from A172 cells was detected in GFP-positive SH-SY5Y cells expressing the P301L mutation as well as in control cells (indicated by the white arrows), in both direct and indirect co-culture conditions (original pictures, **left panels**). The presence of transferred mitochondria within recipient cells was further confirmed through 3D reconstruction using Imaris software (3D: **center panels**, 3D rotated: **right panel**). SH-SY5Y cells are shown in grey, astrocytic mitochondria in magenta. The 3D renderings were rotated relative to the original images to better visualize mitochondrial internalization.

SH-SY5Y GFP-tagged cells (control or P301L) were co-cultured with A172 cells expressing RFP-tagged mitochondria for 24 to 48 h. Co-culture was performed either in direct contact or indirectly using 3 µm inserts. Mitochondrial transfer was assessed qualitatively by fluorescence microscopy and quantitatively by flow cytometry (Figure 1A).

After 24 h of co-culture, we observed a mitochondrial transfer from astrocytes to neurons in both healthy and pathological conditions (Figure 1B). The transfer occurred in both direct and indirect co-culture settings, suggesting that at least some of the transfer occurs via extracellular vesicles or release of free mitochondria by astrocytes. To verify mitochondrial internalization, z-stack images were reconstructed in 3D using Imaris. The 3D rendering confirmed that the transferred mitochondria were located inside recipient cells and not being surface-bound (Figure 1B) (Supplementary Movies S1–S4).

### 3.2. Mitochondrial Transfer from Astrocytes to Neurons Is Increased in P301L Cells

**Figure 2:**
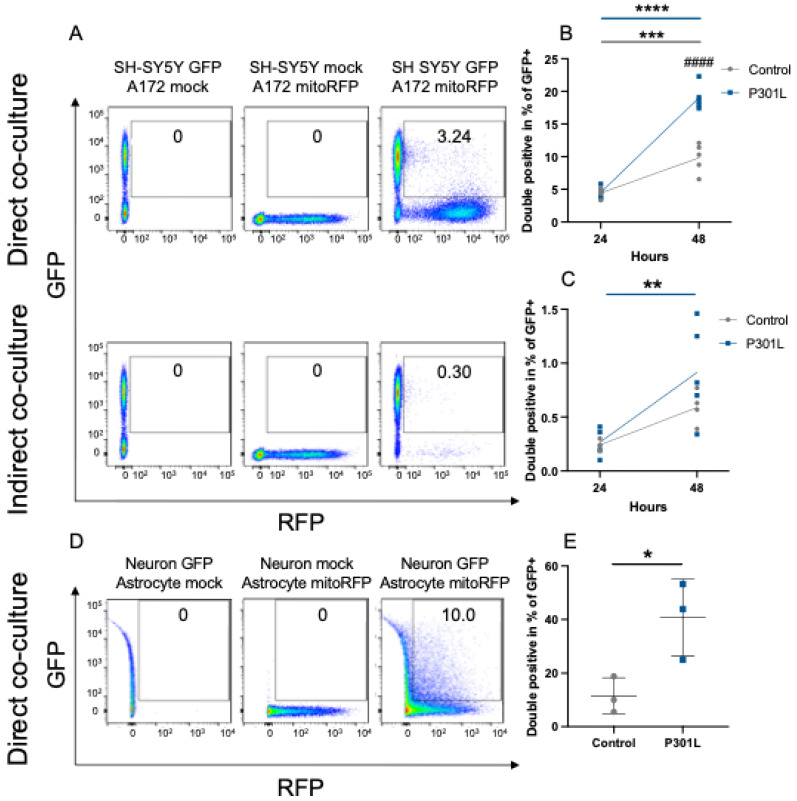
Quantitative assessment of mitochondrial transfer. (**A**) Representative dot plots illustrating the gating strategy for both direct (top) and indirect (bottom) co-culture conditions. MitoRFP-positive events within the GFP-positive SH-SY5Y population (top right quadrant in the dot plots) show the population of SH-SY5Y that received mitochondria from A172 astrocytes. Mitochondrial transfer was assessed after 24 and 48 h of direct (**B**) and indirect (**C**) co-culture in both control (grey) and P301L (blue) cell lines. Dots represent data from independent experiments. Statistical significance was assessed with two-way ANOVA test followed by Sídák’s multiple comparisons test in order to compare the control and P301L group at 24 and 48 h of co-culture. “*” Indicates a significant difference between the 24 h and 48 h conditions (blue bars: P301L cells, grey bar: control). *p* < 0.01 (**), *p* < 0.001 (***), *p* < 0.0001 (****). “####” indicates a significant difference between control and P301L conditions, *p* < 0.0001. *n* = 4–5 independent experiments. (**D**) Representative dot plots illustrating the gating strategy used with co-cultures of iPSC-derived neurons and astrocytes. (**E**) Mitochondrial transfer after 48 h of co-culture between neurons and astrocytes derived from iPSC. Data are presented as mean ± SD and include all biological replicates. Statistical significance was assessed with an unpaired *t*-test. *p* < 0.05 (*). *n* = 3 independent experiments.

Because IMT occurs in both healthy and pathological conditions and appears to involve multiple transfer pathways, we aimed to quantify the proportion of mitoRFP in our SH-SY5Y GFP-positive cells using either a contact-dependent or a non-contact-dependent pathway (Figure 2).

IMT quantification by flow cytometry revealed that after 24 h of direct co-culture, the proportion of mitochondrial transfer from A172 to SH-SY5Y was comparable between control and P301L cells, with 4.4% and 4.5% of neuronal cells containing astrocytic mitochondria, respectively (Figure 2B). In the indirect co-culture setting, only 0.24% of control cells and 0.26% of P301L cells contained astrocytic mitochondria, with no statistically significant difference between the control and P301L cells (Figure 2C).

After 48 h of co-culture, we observed a significant increase in mitochondrial transfer in both control and P301L cells under direct co-culture conditions (Figure 2B), whereas under indirect co-culture conditions, this increase was significant only in P301L cells (Figure 2C). Quantitative analysis revealed that, following 48 h of direct co-culture, a higher proportion of P301L cells (19.0%) had acquired astrocytic mitochondria compared with control cells (9.8%) (Figure 2B). In contrast, under indirect co-culture conditions, no statistically significant difference was detected between control (0.59%) and P301L (0.91%) Figure 2C).

Interestingly, the non-contact-dependent transfer accounts for only a small fraction of total mitochondrial transfer. Indeed, based on our data after 24 h of co-culture, non-contact-dependent transfer represented 5.4% of total IMT in control cells and 5.9% in P301L cells. After 48 h, these values were 6.0% in control cells and 4.8% in P301L cells.

To validate these findings in a more physiologically relevant model, we used patient-derived iPSCs bearing the P301L mutation (P301L cells) or the isogenic CRISPR-Cas9-edited wild-type (WT, Control) cells. We established a direct co-culture of iPSC-derived astrocytes (Control) and neurons (Control or P301L) for 48 h. Consistent with our observations in cell lines, we detected a significant increase in mitochondrial transfer in P301L cells under these conditions. Together, these findings suggest that IMT is increased in a context of tauopathy and that its predominant pathway is contact-dependent.

### 3.3. The Number of Tunneling Nanotubes Is Increased Between Astrocytic and P301L Cells

**Figure 3:**
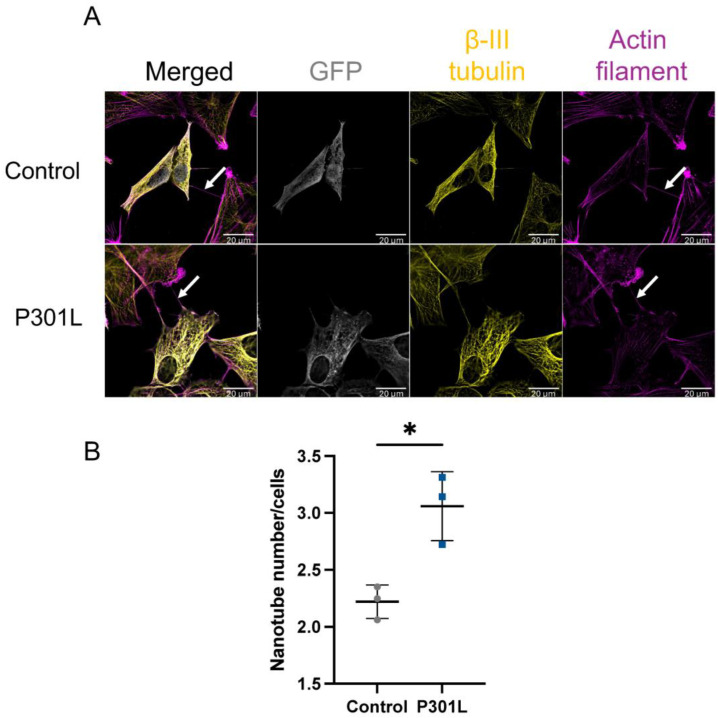
Quantification of tunneling nanotubes between SH-SY5Y and A172 cells. (**A**) After 48 h of co-culture of A172 cells with GFP-expressing SH-SY5Y cells carrying the P301L mutation (P301L) or not (control), actin filaments and βIII-tubulin were stained to distinguish tunneling nanotubes (indicated by the white arrow) from neurites. SH-SY5Y cells are shown in grey, βIII-tubulin in yellow, and actin filaments in magenta (false colors). Only actin-based structures connecting an A172 cell to an SH-SY5Y cell and not colocalizing with βIII-tubulin were included in the count. (**B**) Scatter plot showing the mean number of tunneling nanotubes per cell (±SD). Each dot represents one independent experiment (*n* = 3), calculated from 17–26 control cells and 14–25 P301L cells per experiment. Statistical significance was assessed with the Welch’s *t*-test, *p* < 0.05 (*).

Considering that IMT from astrocytic to neuronal cells was increased in the presence of P301Ltau, we sought to gain physiological insight into the mechanisms underlying this effect. Given that the main pathway of transfer is contact-dependent, we quantified the number of TNTs after 48 h of co-culture, the time point at which a difference between the two conditions was observed (Figure 3A).

We found that the number of TNTs per SH-SY5Y cell was significantly higher in P301L cells compared with control cells. In addition, P301L cells exhibited a higher maximum number of TNTs per cell compared with control cells (Figure 3B).

### 3.4. Fate of the Transferred Mitochondria in Recipient Cells

**Figure 4:**
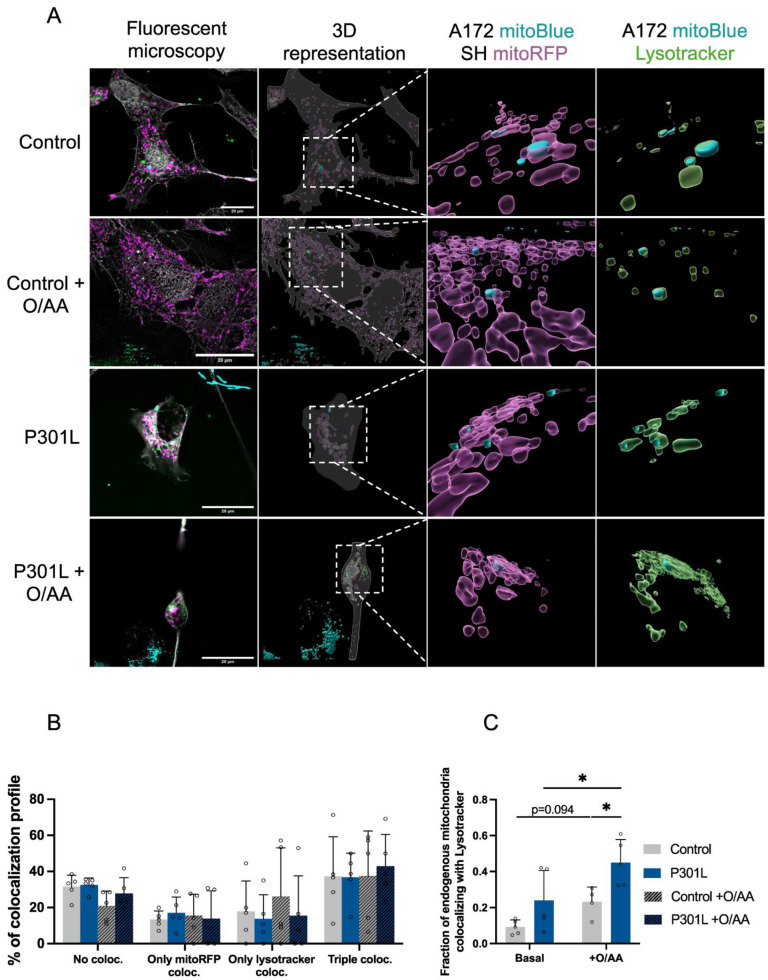
Assessment of the fate of donor mitochondria in the recipient cells. (**A**) Representative image and 3D reconstruction illustrating the fate of transferred mitochondria (false-colored) in SH-SY5Y bearing the mutation P301L or not (control). Mitophagy was induced with Oligomycin (O) and Antimycin A (AA) (+O/AA). SH-SY5Y cells are shown in gray, with their endogenous mitochondria in magenta. Donor mitochondria from A172 cells are shown in cyan, and lysosomes stained with LysoTracker appear in green. (**B**) Bar graph representing the distribution of donor mitochondria across four distinct fate profiles in recipient cells (Control: grey, P301L: blue, Control with induced mitophagy: hatched grey, P301L with induced mitophagy: hatched blue), based on colocalization with mitochondrial and lysosomal markers. “No coloc.” represents the number of SH-SY5Y cells in which mitoBFP2 does not colocalize with either mitoRFP or LysoTracker. “mitoBFP2 × mitoRFP” represents the number of SH-SY5Y cells where mitoBFP2 colocalizes exclusively with mitoRFP. “mitoBFP2 × LysoTracker” represents the number of SH-SY5Y cells where mitoBFP2 colocalizes exclusively with LysoTracker. “Triple coloc.” refers to SH-SY5Y cells in which mitoBFP2 colocalizes with both mitoRFP and LysoTracker. Statistical significance was assessed with three-way ANOVA followed by Tukey’s multiple comparisons test, in order to compare the different colocalization profiles depending on the cell type and the induction or not of mitophagy. Data are represented as mean ± SD. Each dot represents one independent experiment (*n* = 5), with values corresponding to the mean of all analyzed cells within that experiment. Under basal conditions, 13–35 control cells and 14–31 P301L cells were analyzed per experiment. Under mitophagy-inducing conditions, 7–22 control cells and 10–17 P301L cells were analyzed per experiment. (**C**) Bar graph representing the fraction of endogenous mitochondria colocalizing with lysotracker signal. Data are represented as mean ± SD. Each dot represents one independent experiment (*n* = 5), with values corresponding to the mean of all analyzed cells within that experiment. Under basal conditions, 12–22 control cells and 14–29 P301L cells were analyzed per experiment. Under mitophagy-inducing conditions, 10–36 control cells and 9–22 P301L cells were analyzed per experiment. Statistical significance was assessed with a two-way ANOVA assay, *p* < 0.05 (*).

Since IMT occurs from astrocytic to neuronal cells and is exacerbated in abnormal tau conditions, we sought to determine the fate of transferred mitochondria in recipient cells. We performed a 48 h direct co-culture between SH-SY5Y cells (control or P301L) expressing GFP and mitoRFP, and A172 cells expressing mitoBFP2. Lysosomes were stained to assess whether donor-derived mitochondria were degraded in recipient cells rather than incorporated into the mitochondrial network. Because abnormal tau protein is known to affect mitophagy, we also induced mitophagy using oligomycin and antimycin A (O/AA) to determine whether the differences observed between P301L and control conditions are specifically driven by pathological P301Ltau expression (Figure 4A). Interestingly, we identified four distinct populations of SH-SY5Y recipient cells based on the colocalization profile of mitoBFP2 from A172 with endogenous mitochondria (mitoRFP) and lysosomes (LysoTracker). Under basal conditions, both pathological and control conditions show a similar proportion of SH-SY5Y cells (32.4% and 31.5%, respectively) containing mitoBFP2-positive mitochondria that did not colocalize with either marker, suggesting incomplete integration or a transitional state (Figure 4B). A subset of cells showed exclusive colocalization of donor mitochondria with mitoRFP (13.4% in control cells and 17.1% in P301L-expressing cells) or with LysoTracker (17.8% in control cells and 13.8% in P301L SH-SY5Y cells). Most cells displayed colocalization with both markers, indicating a mixed fate of donor mitochondria. This profile was observed in 37.2% of control cells and 36.8% of P301L cells. However, no significant difference was observed in the proportion of colocalization profiles between the two cell types.

As in basal conditions, no significant difference was observed between control and P301L cells in the colocalization profile under induced mitophagy with oligomycin and antimycin A. Overall, no colocalization profile was detected in 20.9% of control cells compared with 27.8% of P301L cells. Donor mitochondria showed exclusive colocalization with mitoRFP in 15.6% of control cells versus 13.8% of P301L cells. Exclusive colocalization between mitoBlue and LysoTracker was observed in 26.1% of control cells and 15.5% of P301L cells. Finally, triple colocalization was detected in 37.4% of control cells compared with 42.9% of P301L cells (Figure 4B).

To verify the effectiveness of the oligomycin/antimycin A treatment, we quantified the colocalization between endogenous SH-SY5Y mitochondria (mitoRFP) and LysoTracker under basal and mitophagy-inducing conditions (+O/AA). As expected, O/AA treatment enhanced mitophagy in both cell lines (Figure 4C). Interestingly, we also noticed that under mitophagy-inducing conditions, P301L cells exhibited a significantly higher level of mitochondria–lysosome colocalization compared with control cells, suggesting increased mitophagy when P301L cells are co-cultured with A172 cells.

Overall, these data indicate that in both control and P301L cells, the transferred astrocytic mitochondria can fuse with the endogenous mitochondrial network and/or be degraded in lysosomes. In addition, co-culturing SH-SY5Y cells with A172 cells significantly increased the degradation of endogenous mitochondria in the P301L cells compared with the control cells.

### 3.5. Mitochondrial Transfer Increases Cells’ Bioenergetics

**Figure 5:**
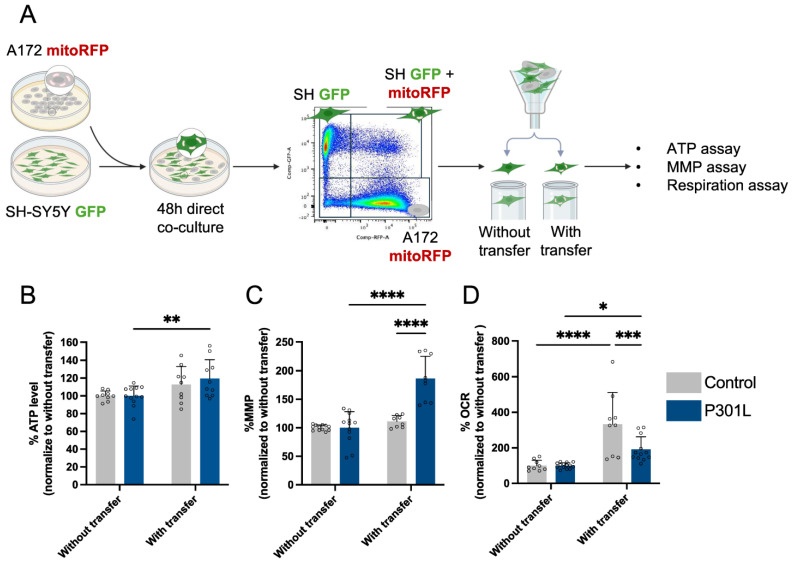
Effect of mitochondrial transfer on recipient cells. (**A**) Schematic representation of the experimental design. SH-SY5Y GFP cells (green cells) were co-cultured with A172 cells expressing RFP-labeled (grey cells) mitochondria for 48 h. SH-SY5Y cells that had received mitochondria from A172 and those that had not were subsequently isolated by cell sorting. ATP, mitochondrial membrane potential (MMP), and respiration assays were then performed on the sorted populations. (**B**) Bar graph representing ATP level in SH-SY5Y control (grey) and P301L (blue) cells with or without transferred mitochondria. (**C**) Bar graph representing MMP level in SH-SY5Y control (grey) and P301L (blue) cells with or without transferred mitochondria. (**D**) Bar graph representing the oxygen consumption rate (OCR) values derived from the area under the curve of the respiration profile in SH-SY5Y control (grey) and P301L (blue) cells with or without transferred mitochondria. All data were normalized to the “without transfer” condition and are presented as mean ± SD. In addition, each replicate is represented as a circle. Statistical significance was assessed with a two-way ANOVA. *p* < 0.05 (*), *p* < 0.01 (**), *p* < 0.001 (***), *p* < 0.0001 (****). *n* = 3 independent experiments with 3–4 replicates per experiment.

Based on our findings, we investigated the impact of mitochondrial transfer on the bioenergetics of recipient cells compared with cells that did not receive astrocytic mitochondria. SH-SY5Y GFP cells (control or P301L) were co-cultured with A172 mitoRFP cells for 48 h. Cells were then sorted to isolate SH-SY5Y cells that had either received mitoRFP from astrocytes or not, and subsequently subjected to ATP, MMP, and respiration assays. As both populations were derived from the same co-culture, this experimental design allowed the specific assessment of mitochondrial transfer effects independently of co-culture-related factors (Figure 5A).

In control cells, mitochondrial transfer did not significantly increase ATP production or mitochondrial membrane potential (MMP) (Figure 5B,C), despite a marked increase in cellular respiration (+285.9%; Figure 5D). In contrast, in P301L cells, mitochondrial transfer resulted in a significant increase in ATP levels (+19.4%), MMP (+86.1%), and respiration (+91.3%) (Figure 5B–D). Notably, the increase in respiration was significantly more pronounced in control cells than in P301L cells. Conversely, MMP levels were significantly higher in P301L cells compared with control cells following mitochondrial transfer.

These data suggest that mitochondria transferred from A172 to SH-SY5Y cells expressing P301Ltau improve cellular bioenergetics in P301L cells, as evidenced by a significant increase in ATP levels, MMP, and cellular respiration.

## 4. Discussion

Intercellular mitochondrial transfer is now a well-established phenomenon. In vivo imaging in zebrafish has enabled real-time visualization of mitochondrial transfer via TNTs [33]. In mice, satellite glial cells have been shown to similarly transfer mitochondria to sensory neurons through TNTs, as shown by in vivo tracking of fluorescently labeled mitochondria [34]. Together, these findings support the physiological relevance of IMT in vivo. However, how pathological conditions, particularly tauopathy, influence this process remained poorly understood. To our knowledge, our study is the first to show that P301L-tau protein is associated with increased astrocyte-to-neuron mitochondrial transfer, thereby improving the bioenergetics of recipient cells. Additionally, we showed that this transfer is associated with an increase in the number of TNTs between P301L-expressing neuronal cells and astrocytes, as well as increased mitophagy.

Abnormal tau protein is known to impair both actin filament dynamics and mitochondrial function, including transport [1,4]. To study the effects of abnormal tau protein on IMT, we used differentiated SH-SY5Y cells bearing the P301L tau mutation, a cellular model previously characterized in our laboratory, which displays mitochondrial dysfunctions consistent with those described in the literature [35,36]. Neurons and astrocytes derived from IPCSs were also used to confirm the impact of abnormal tau protein on IMT. We hypothesized that pathological tau may also impair mitochondrial transfer between cells due to its involvement in the disruption of actin filament dynamics. Our results reveal that mitochondrial transfer was increased in the tauopathy condition, which aligns with literature showing that pathological conditions and cellular stress stimulate IMT. In our model, the non-contact-dependent transfer accounted for only a minor fraction of total IMT, suggesting that contact-dependent transfer pathways, such as tunneling nanotubes and gap junctions, may play a dominant role in IMT from astrocytes to neurons. Consistent with our data, we observed an increased number of TNTs in P301L cells compared with control cells. This increase was associated with higher IMT levels observed in the pathological condition, although the present study does not establish a causal relationship between TNT formation and mitochondrial transfer. Interestingly, this association is consistent with the current literature describing the impact of pathological tau on actin dynamics. Indeed, abnormal tau has been shown to increase F-actin levels, a critical structural component of TNTs [37]. Since these protrusions rely on an F-actin scaffold, abnormal tau protein-mediated polymerization and inhibited depolymerization [37] may contribute to the increased abundance of TNTs observed in P301L cells. However, further studies will be required to determine whether these changes directly influence IMT.

Some factors could explain the preferential use of TNTs/gap junctions over vesicle-mediated transfer under pathological conditions. Previous studies have shown that, in response to cellular stress, injured or energy-deficient cells can emit signals that induce the formation of TNTs, which are initiated by the stressed cell and directed toward a neighboring supportive cell [28,38]. This mechanism could provide a more specific, direct, and faster transfer between two cells than with EVs, while also avoiding exposure of the transferred mitochondria to oxidative stress or lysosomal enzymes present in the extracellular environment. Indeed, it has been shown that non-contact-dependent transfer not only involves vesicular transport but also includes passive release of mitochondrial material into the extracellular space [17]. The ability of stressed or damaged cells to initiate TNT formation as a “help signal” may therefore contribute to the increased IMT observed in our tauopathy model. One hypothesis is that the abnormal tau protein indirectly promotes mitochondrial transfer by impairing mitochondrial function, thereby inducing cellular stress and triggering TNT formation. However, despite growing interest in TNTs, the molecular mechanisms governing their formation remain poorly understood. Consequently, it is currently difficult to determine the precise mechanisms responsible for the increase in IMT observed in the context of tau pathology. Further studies will therefore be required to elucidate how pathological tau influences TNT formation and regulates IMT.

Given the observed increase in IMT from A172 to SH-SY5Y in the context of tauopathy, we initially hypothesized that a greater proportion of transferred mitochondria would integrate into the mitochondrial network of SH-SY5Y cells expressing the P301L mutation as a compensatory response to tau-induced mitochondrial dysfunction. The analysis of colocalization profiles revealed that transferred astrocytic mitochondria adopt multiple fates within recipient SH-SY5Y cells, including colocalization with the endogenous mitochondrial network, degradation via lysosomal pathways, or persistence as non-integrated organelles. Notably, these distributions were comparable between control and P301L cells under both basal and induced mitophagy conditions, indicating that the presence of pathological tau does not seem to alter the intracellular processing of transferred mitochondria. The coexistence of mitochondria colocalizing with both mitoRFP and LysoTracker further supports a dynamic balance between mitochondrial fusion and degradation, suggesting ongoing quality control mechanisms. Under mitophagy-inducing conditions, a slight shift toward increased triple colocalization in P301L cells may reflect enhanced mitochondrial turnover, although this did not reach statistical significance.

Pathological tau is known to impair mitochondrial quality control and mitophagy [15]. Surprisingly, we observed significantly higher mitochondrial–lysosomal colocalization in P301L cells than in control cells under mitophagy-inducing conditions. One possible explanation is that the co-culture with the astrocytic cell line partially restores mitochondrial quality control mechanisms in P301L cells. This beneficial effect may result from the transfer of healthy mitochondria from the A172 cells, a proportion of which colocalize with endogenous mitochondria, thereby contributing to the recipient mitochondrial network. Alternatively, or in addition, A172 cells may release soluble factors that enhance mitochondrial homeostasis and lysosomal function in recipient neurons. Although the underlying mechanisms remain to be determined, these findings suggest that astrocyte–neuron interactions may enhance P301L cells’ capacity to induce mitophagy under stress conditions.

We next assessed the functional consequences of mitochondrial transfer on the bioenergetic profile of recipient SH-SY5Y cells. Our results demonstrate that the acquisition of astrocytic mitochondria enhances cellular respiration in both control and P301L cells, indicating that transferred mitochondria are functionally active within recipient cells. Interestingly, although this increase in respiration was more pronounced in control cells, it did not translate into measurable increases in ATP and MMP levels. As mentioned previously, P301L cells exhibit impaired mitochondrial function (lower respiration, ATP and MMP levels) [1,4]. This pre-existing dysfunction may explain their more limited increase in respiration following mitochondrial transfer compared with control cells. In contrast, control cells are likely already operating at a relatively optimal bioenergetic state, with sufficient ATP production and MMP. Consequently, the additional mitochondria acquired through transfer may enhance respiratory capacity without producing a measurable increase in ATP levels or membrane polarization. This suggests that the additional respiratory activity may reflect an increase in metabolic capacity rather than a direct enhancement of energetic efficiency. In contrast, P301L cells exhibited a significant increase in MMP and ATP levels following mitochondrial transfer, indicating a potential partial restoration of bioenergetic function in this pathological context. However, quantification of neuronal mitochondrial mass would further strengthen these findings by clarifying whether the observed bioenergetic improvement is associated with changes in mitochondrial content. Moreover, additional analyses will be required to fully characterize the impact of transferred mitochondria on mitochondrial function.

The bioenergetic benefit of IMT in recipient cells has been previously demonstrated. For example, Baldwin et al. (2024) reported mitochondrial transfer from bone marrow stromal cells to CD8+ T lymphocytes in co-culture, resulting in enhanced bioenergetic function in recipient cells [39]. However, to our knowledge, we are the first to demonstrate such a beneficial effect in the context of tauopathy and to assess how pathological tau influences this transfer.

We acknowledge that our study presents some limitations. Indeed, the majority of the experiments were performed using established cell lines which may not fully recapitulate the complexity of IMT in the brain. Nevertheless, the increase in IMT observed in the P301L condition was detected in both cell lines and neurons derived from IPSCs. This concordance supports the relevance of our primary experimental model and suggests that the observed phenomenon is not restricted to immortalized cell lines.

The present work also focused exclusively on IMT from astrocytes to neurons and did not investigate the reciprocal transfer from neurons to astrocytes. Bidirectional mitochondrial exchange has been reported in the central nervous system and is also known to participate to the spread of abnormal tau protein to other brain cells [40]. The primary objective of this study was specifically to investigate the supportive role of astrocytes toward neurons in the context of tau pathology. This rationale was based on prior work from our laboratory demonstrating that astrocyte mitochondrial transplantation confers beneficial effects on P301L cells [41]. Future studies should therefore explore the functional consequences of bidirectional mitochondrial transfer between neurons and astrocytes in tauopathies.

Overall, our study identifies IMT as a compensatory bioenergetic response in the context of tauopathy. We showed that this predominantly contact-dependent process exerts beneficial effects on cellular bioenergetics, supporting the idea of developing new mitochondria-based therapeutic strategies. In recent years, mitochondrial transplantation has emerged as a promising approach. For example, a recent study from our group demonstrated that mitochondrial transplantation in neuronal cells expressing the P301L mutation improves cellular bioenergetics and enhances neurite outgrowth[41]. In parallel, other studies on stroke/brain injuries, cancer, cognitive disorders, and neurodegenerative diseases have shown similar positive effects on brain cells [42]. While these results support the promise of mitochondria-based approaches, one should take into account that in a chronic, progressive tauopathy scenario, transplanted or newly acquired exogenous mitochondria will inevitably face the same proteotoxic and pro-oxidant environment as the endogenous pool. Therefore, exogenous organelle integration may offer a temporary bioenergetic window of neuroprotection rather than a permanent metabolic arrest of the disease. Future translational strategies will likely need to explore combined therapeutic regimens that pair mitochondrial transplantation to acutely restore ATP levels and cellular respiration with anti-tau therapies designed to clear the underlying pathological environment.

## 5. Conclusions

In this study, we showed that IMT is an active and adaptive response that is preserved, and even enhanced, in tauopathies. Indeed, P301L cells showed increased TNT formation and higher mitochondrial transfer. Importantly, while tau pathology increases the rate of mitochondrial transfer, it does not appear to significantly alter the intracellular fate of transferred mitochondria, which are similar in both control and P301L cells. Functionally, transferred astrocytic mitochondria are bioenergetically competent and can improve cellular respiration in both contexts, with a significant effect in P301L cells, where ATP levels are significantly increased. Altogether, these findings suggest that IMT serves as a compensatory metabolic adaptation in tauopathy, supporting neuronal bioenergetics, possibly by boosting mitochondrial quality-control pathways in recipient cells.

## Data Availability

The data presented in this study are available on: https://osf.io/zfdga/overview?view_only=91980eea9f0142f291ad6550f670fe5c (accessed on 30 April 2026).

## Supplementary Materials

The following supporting information can be downloaded at: https://www.mdpi.com/article/10.3390/cells15121101/s1.

## Abbreviations

The following abbreviations are used in this manuscript:

AA	Antimycin A
ATP	Adenosine Triphosphate
DMEM	Dulbecco’s Modified Eagle Medium
EVs	Extracellular vesicles
FBS	Fetal Bovine Serum
GFP	Green Fluorescent Protein
HS	Horse Serum
IMT	Intercellular Mitochondrial Transfer
mitoBFP2	Blue Fluorescent Protein 2 -labeled mitochondria
mitoRFP	Red Fluorescent Protein-labeled mitochondria
O	Oligomycin
OCR	Oxygen Consumption Rate
SD	Standard Deviation
TNTs	Tunneling Nanotubes
