# Supplementary material for: Compensatory Intercellular Mitochondrial Transfer Improves Bioenergetics in P301L Tau-Affected Neuronal Cells
Source: Cells. 2026 Jun 17;15(12):1101. doi: 10.3390/cells15121101 (PMC13297239; doi:10.3390/cells15121101)
Supplement: Supplementary file 1 [file cells-15-01101-s001.zip › Supplementary Figures and Movies_proofs.pdf]

Supplementary Figures and Movies:

Supplementary Movie S1: 3D Animation showing mitochondria from A172 mitoRFP cells into SH-SY5Y Control-GFP cells at 24 hours of direct co-culture.
SH-SY5Y Control-GFP cells are shown in gray (GFP fluorescence, false color), and the mitochondria from A172 cells expressing the mitoRFP tag are shown in magenta. The 3D surface reconstruction and animation were generated using Imaris software. The movie corresponds to the image depicted in Figure 1B (Direct co-culture, Control-GFP/A172 Mito-RFP).

Supplementary Movie S2: 3D Animation showing mitochondria from A172 mitoRFP cells into SH-SY5Y P301L-GFP cells at 24 hours of direct co-culture.
SH-SY5Y P301L-GFP cells are shown in gray (GFP fluorescence, false color), and the mitochondria from A172 cells expressing the mitoRFP tag are shown in magenta. The 3D surface reconstruction and animation were generated using Imaris software. The movie corresponds to the image depicted in Figure 1B (Direct co-culture, P301L-GFP/A172 Mito-RFP).

Supplementary Movie S3: 3D Animation showing mitochondria from A172 mitoRFP cells into SH-SY5Y Control-GFP cells at 24 hours of indirect co-culture.
SH-SY5Y Control-GFP cells are shown in gray (GFP fluorescence, false color), and the mitochondria from A172 cells expressing the mitoRFP tag are shown in magenta. The 3D surface reconstruction and animation were generated using Imaris software. The movie corresponds to the image depicted in Figure 1B (Indirect co-culture, Control-GFP/A172 Mito-RFP).

Supplementary Movie S4: 3D Animation showing mitochondria from A172 mitoRFP cells into SH-SY5Y P301L-GFP cells at 24 hours of indirect co-culture.
SH-SY5Y P301L-GFP cells are shown in gray (GFP fluorescence, false color), and the mitochondria from A172 cells expressing the mitoRFP tag are shown in magenta. The 3D surface reconstruction and animation were generated using Imaris software. The movie corresponds to the image depicted in Figure 1B (Indirect co-culture, P301L-GFP/A172 Mito-RFP).

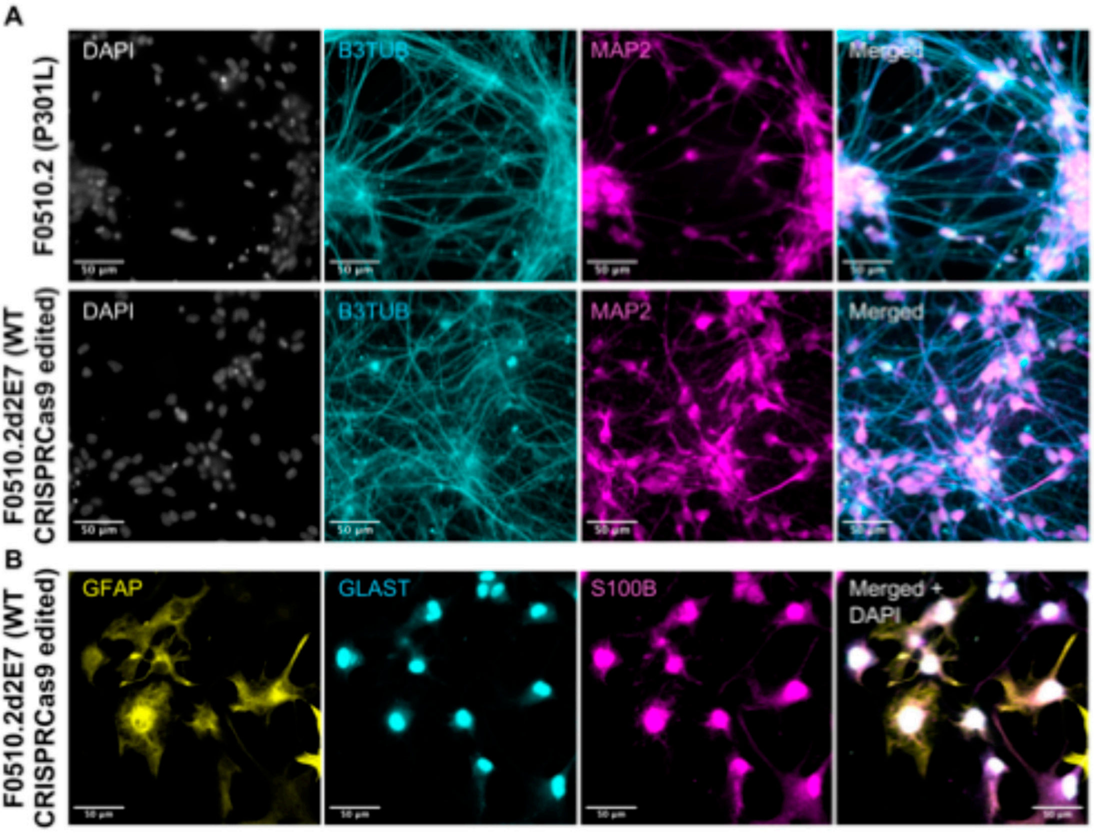

Figure S1: Quality control of iPSC-derived neurons and astrocytes. (A) Quality control of iPSC-derived neurons F0510.2 (P301L) and F0510.2d2E7 (WT). The expression of several neuronal markers was assessed by immunostaining through the staining of B3TUB and MAP2. (B) For iPSC-derived astrocytes we assessed if the cells express the astrocytic markers GFAP, GLAST, and S100B. Scale bars=50 µm. B3TUB: ß3 tubulin, MAP2: Microtubule-Associated Protein 2, GFAP: glial fibrillary acidic protein, GLAST: glutamate aspartate transporter, S100B: S100 calcium-binding protein B

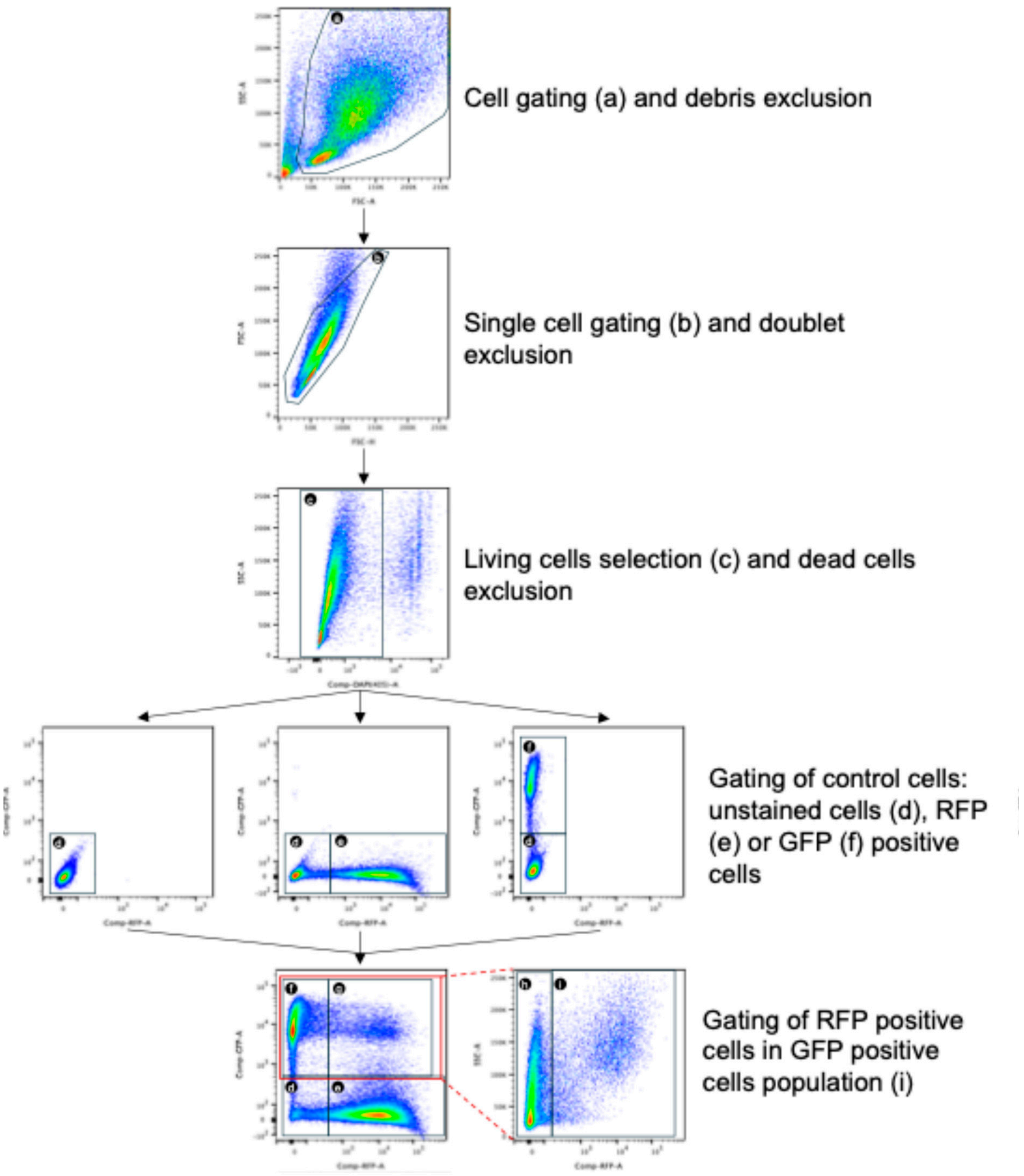

Figure S2: Flow cytometry gating strategy. (a) Cell population was first identified based on forward scatter (FSC) and side scatter (SSC) parameters to exclude debris. (b) Singlets were selected using FSC-A versus FSC-H to remove doublets and cell aggregates. (c) Viable cells were gated by excluding dead cells using the viability dye. (d) Gating of unstained cells usingthe co-culture of SH-SY5Y mock and A172 mock. (e) Gating of RFP positive population using the co-culture of SH-SY5Y mock and A172 mitoRFP (f) Gating of GFP positive population using the co-culture of SH-SY5Y GFP and A172 mock. (g) Gating of the double positive population. To avoid potential bias caused by variations in the double-negative cell population between experiments, we quantified mitochondrial transfer within the entire GFP-positive population (red box) and subsequently determined the proportion of RFP-positive (i) and RFP-negative (h) cells.

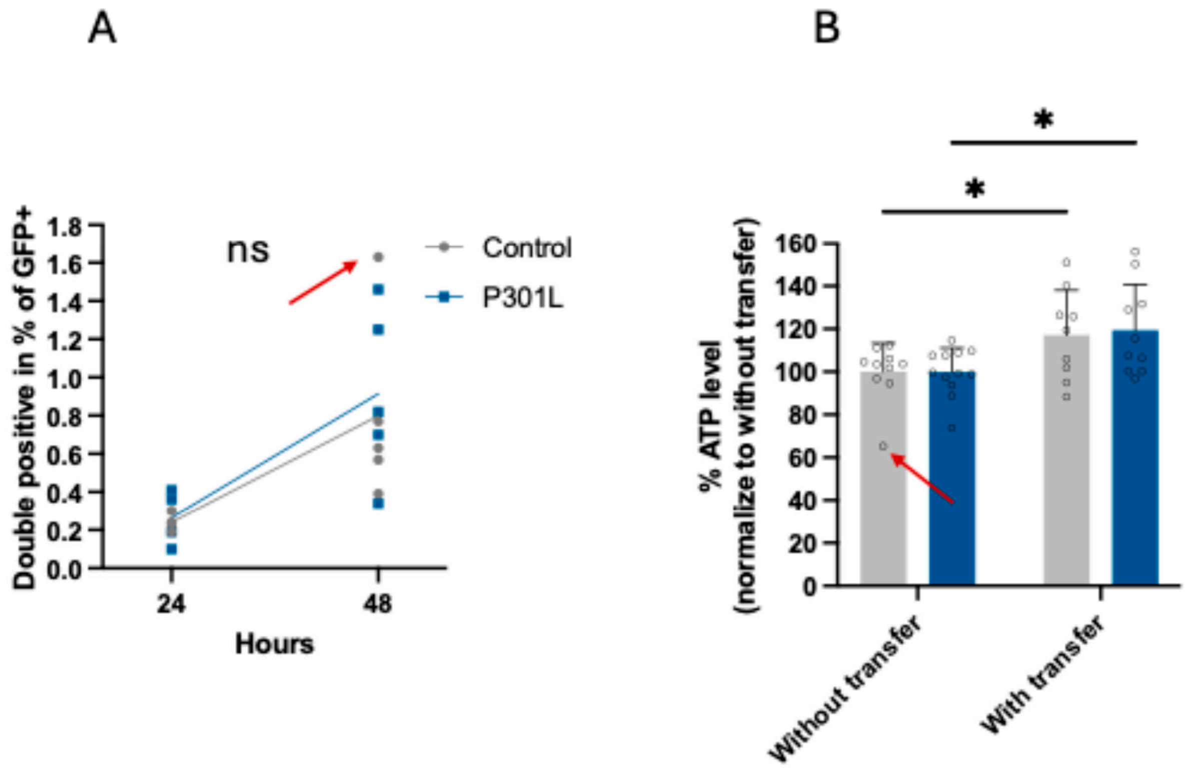

Figure S3: Graphs before outlier removal. (**A**) Bar graph corresponding to Figure 2C prior to outlier exclusion. The data point identified by the red arrow was removed following Grubbs' test. (**B**) Bar graph corresponding to Figure 5B prior to outlier exclusion. The data point identified by the arrow was removed following Grubbs' test Statistical significance was assessed with a two-way ANOVA assay, $p < 0.05$ (*)
